# Sub-Nanometer Cryo-EM Density Map of the Human Heterodimeric Amino Acid Transporter 4F2hc-LAT2

**DOI:** 10.3390/ijms21197094

**Published:** 2020-09-25

**Authors:** Jean-Marc Jeckelmann, Dimitrios Fotiadis

**Affiliations:** Institute of Biochemistry and Molecular Medicine, and Swiss National Centre of Competence in Research (NCCR) TransCure, University of Bern, CH-3012 Bern, Switzerland

**Keywords:** 4F2hc, cryo-EM, heteromeric amino acid transporter, LAT2, L-type amino acid transporter, SLC3, SLC7

## Abstract

Heterodimeric amino acid transporters (HATs) are protein complexes mediating the transport of amino acids and derivatives thereof across biological membranes. HATs are composed of two subunits, a heavy and a light chain subunit belonging to the solute carrier (SLC) families SLC3 and SLC7. The human HAT 4F2hc-LAT2 is composed of the type-II membrane N-glycoprotein 4F2hc (SCL3A2) and the L-type amino acid transporter LAT2 (SLC7A8), which are covalently linked to each other by a conserved disulfide bridge. Whereas LAT2 catalyzes substrate transport, 4F2hc is important for the successful trafficking of the transporter to the plasma membrane. The overexpression, malfunction, or absence of 4F2hc-LAT2 is associated with human diseases, and therefore, this heterodimeric complex represents a potential drug target. The recombinant human 4F2hc-LAT2 can be functionally overexpressed in the methylotrophic yeast *Pichia pastoris*, and the protein can be purified. Here, we present the cryo-EM density map of the human 4F2hc-LAT2 amino acid transporter at sub-nanometer resolution. A homology model of 4F2hc-LAT2 in the inward-open conformation was generated and fitted into the cryo-EM density and analyzed. In addition, disease-causing point mutations in human LAT2 were mapped on the homology model of 4F2hc-LAT2, and the possible functional implications on the molecular level are discussed.

## 1. Introduction

Amino acid transporters play vital roles to provide cells with important substrates as amino acids and derivatives thereof. These transporters belong to different solute carrier (SLC) families of which the SCL7 family comprises 15 members [1]. Members from the SLC7 family also belong to the amino acid, polyamine, and organocation (APC) superfamily of transporters (transport classification (TC) system no. 2.A.3; http://www.tcdb.org [2]) and are further subdivided into two groups, i.e., the cationic amino acid transporters (CATs; SLC7A1–4 and SLC7A14) and the L-type amino acid transporters (LATs; SLC7A5-A11, Slc7a12, SLC7A13, and Slc7a15). Whereas CATs are N-glycosylated, LATs are not. Instead, they associate with glycoproteins from the SLC3 family, e.g., 4F2hc (SLC3A2, CD98) or rBAT (SLC3A1), to form heterodimers. In these complexes, LATs are referred as the light chains and the SLC3 family proteins are referred as the heavy chains. The two components of these heterodimeric amino acid transporters (HATs) are covalently linked by a conserved disulfide bridge [1,3,4]. Whereas the light chains, i.e., the LATs, are the catalytic part of HATs, heavy chains stabilize the complex and are responsible for their correct trafficking to the plasma membrane [5,6]. The HAT 4F2hc-LAT2 (SLC3A2-SLC7A8) is an obligatory exchanger. It preferably transports neutral as well as small L-amino acids [7,8,9] and the thyroid hormones T_3_ and T_4_ [10] in a sodium-independent manner [8]. 4F2hc is a type II membrane N-glycoprotein that consists of a small N-terminal cytoplasmic-domain, a single transmembrane helix (TM), and a large C-terminal ectodomain (ED) [1,11]. Although bacterial glucosidases are similar in structure to 4F2hc-ED, the latter does not possess glucosidase activity [12]. The light subunit LAT2 is not glycosylated, highly hydrophobic, and predicted to contain 12 transmembrane domains with cytoplasmic N- and C-termini [1,8]. From a medical point of view, LAT2 is expressed in several tissues as the brain, heart, small intestine, lung, spleen, liver, prostate, kidney, ovary, testis, skeletal muscle, placenta, and fetal liver [1]. Whereas the loss of LAT2-dependent transport function is attributed to age-related hearing loss, renal aminoaciduria, and cataract formation [1,13,14,15], an elevated expression level of LAT2 is linked to cancer [16].

The methylotrophic yeast *Pichia pastoris* is widely used as a recombinant protein expression host [17]. The system is particularly useful for the expression of membrane proteins for structural studies [18,19]. After an overexpression screening in *P. pastoris* including nine different heavy and light chains, the human HAT 4F2hc-LAT2 was identified as a promising target for functional and structural studies [5,20,21,22]. Overexpression followed by affinity purification yielded a functional, recombinant N-terminal His- (4F2hc) and Strep-tagged (LAT2) heterodimeric complex 4F2hc-LAT2 correctly connected by the conserved disulfide bridge [5,21,22]. The amino acid transport activity of the recombinant human complex was either shown by L-leucine uptake into proteoliposomes or whole cell uptake experiments using Pichia cells [5,20]. Structures of 4F2hc-ED and 4F2hc-LAT1 were solved by X-ray crystallography [12] and cryogenic electron microscopy (cryo-EM) [23,24], respectively. Currently, there is no high-resolution structure of 4F2hc-LAT2 available. The 4F2hc-LAT2 volumes published so far display a continuous increase in resolution from ≈20 Å to 13 Å, which is mainly attributed to the improvement of the protein purification strategy in terms of detergent usage and the electron microscopy techniques applied [5,21,22].

Here, we report on the cryo-EM density map of the glyco-diosgenin (GDN) micelle embedded human 4F2hc-LAT2 complex at sub-nanometer resolution. A comparison of side-view 2D-class averages of 4F2hc-LAT2 with the back-projection of the published 4F2hc-LAT1 density revealed an inward-open conformation of 4F2hc-LAT2 particles. This stimulated us to generate a homology model of the human 4F2hc-LAT2 exchanger based on the coordinates of human LAT1. This homology model fitted nicely into the 4F2hc-LAT2 cryo-EM 3D density map. Furthermore, the 4F2hc-LAT2 homology model was also used to map disease-related point mutations of the heterodimeric complex, giving insights into the possible causes that compromised the transport function of 4F2hc-LAT2 mutants on a molecular basis.

## 2. Results and Discussion

**Figure 1:**
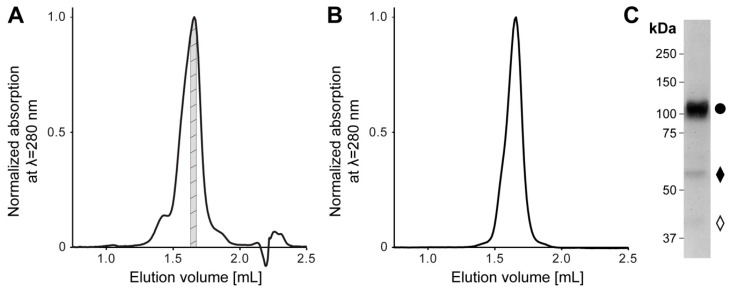
Size exclusion chromatography (SEC) and SDS-PAGE analysis of purified human 4F2hc-LAT2 complex. (**A**) Representative SEC elution profile of nickel nitrilotriacetic acid (Ni-NTA) purified 4F2hc-LAT2 complex using a Superose 6 Increase 3.2/300 column. A major elution peak at 1.66 mL flanked by two minor peaks is discerned. The gray highlighted protein fraction was analyzed toward its stability and purity by a second SEC run (B) and SDS-PAGE (C) or used directly for cryogenic electron microscopy (cryo-EM) grid preparation. (**B**) Analysis of the cryo-EM 4F2hc-LAT2 sample by SEC displays a prominent and almost symmetrical elution peak free from aggregates or lower molecular weight entities. The peak elutes at about the same elution volume as in panel (A), indicating protein complex stability and monodispersity. (**C**) SDS-PAGE (8% gel, silver staining, 2 µg sample loaded) analysis of the SEC-purified heterodimeric human 4F2hc-LAT2 complex (●) sample used for cryo-EM grid preparation displays a high sample purity. Faster migrating bands are composed of 4F2hc (♦) and LAT2 (◊). LAT: L-type amino acid transporter.

Recombinant human 4F2hc-LAT2 complex was overexpressed using the methylotropic yeast *P. pastoris*, and affinity chromatography was purified as described previously [5,20,21,22]. Isolated membranes were solubilized in lauryl maltose neopentyl glycol (LMNG)/cholesteryl hemisuccinate (CHS) and bound to nickel nitrilotriacetic acid (Ni-NTA) affinity chromatography resin. During the affinity purification procedure, the detergent was exchanged from LMNG/CHS to the steroid-based glyco-diosgenin (GDN) detergent. The concentrated eluate was subjected to size exclusion chromatography (SEC). The elution profile showed a major peak flanked by two minor ones on the left and right side (Figure 1A). The major SEC peak fraction was analyzed regarding its stability and purity by two different methods, i.e., a second SEC-run and SDS-polyacrylamid gel electrophoresis (SDS-PAGE) (Figure 1B,C). In comparison to the first, the SEC elution profile of the second SEC-run displayed an almost symmetrical peak free from aggregates or lower molecular weight entities (Figure 1A,B). Furthermore, SDS-PAGE confirmed the stability and high purity of the recombinant heterodimeric protein complex purified by SEC. As previously reported, the minor fraction of faster migrating bands compared to the covalently linked heterodimeric complex 4F2hc-LAT2 (Figure 1C) were attributed to 4F2hc and LAT2 [20]. We hypothesize that this minor amounts of free 4F2hc and LAT2 arise upon SDS denaturation and/or the SDS-PAGE of the complex, since the second SEC elution profile (Figure 1B) displayed only one major peak, which was free from lower molecular weight entities. In summary, the affinity and SEC purified 4F2hc-LAT2 complex embedded in a GDN micelle was correctly assembled, stable, and of high purity and homogeneity, thus representing an ideal candidate for further structural studies.

**Figure 2:**
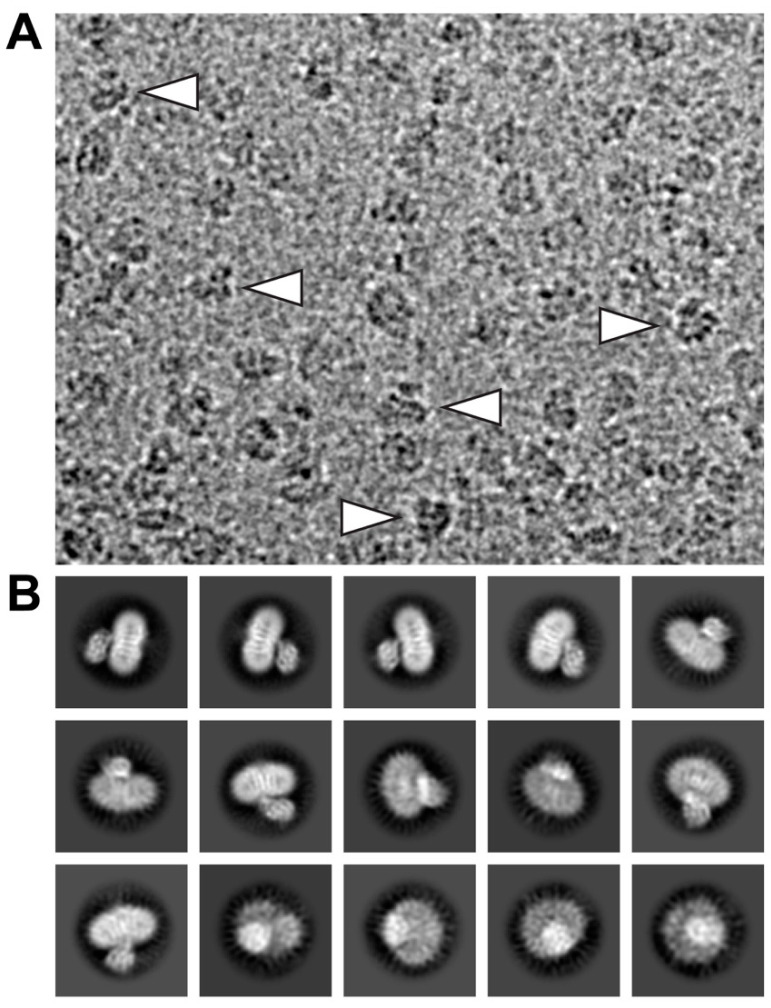
Electron micrograph and 2D-class averages of glyco-diosgenin (GDN) purified 4F2hc-LAT2 particles. (**A**) Shown is a representative cryo-EM image collected using a Titan Krios G4 electron microscope recorded at −2.0 µm defocus. The 4F2hc-LAT2 particles are homogenously distributed, viewed from different angles, and approximately 100-120 Å in size. Indicated with white arrowheads are particle projections as viewed from the side, displaying the characteristic bilobed structure and a strong micellar density. (**B**) Displayed is a gallery of 15 representative 2D-class averages, which are ordered according to the decreasing number of particles assigned to each class. The protein density is black and white in panels (**A**) and (**B**), respectively. Frame sizes of individual pictures are 180 nm × 140 nm (**A**) and 230 Å (**B**).

Electron micrographs of SEC-purified and GDN-embedded 4F2hc-LAT2 particles were recorded on a high-end 300 kV Thermo Fisher Scientific Titan Krios G4 electron microscope equipped with a Falcon 4 direct electron detector camera. Cryo-EM images revealed a homogenous particle distribution showing a bilobed 4F2hc-LAT2 particle shape when viewed from the side (Figure 2A, arrowheads) and displayed a very intense density in the micellar headgroup region. Examples of representative 2D-class averages of 4F2hc-LAT2 particles show projections as viewed from different angles (Figure 2B). Despite the intense micellar density on the particle level, the transmembrane helices (TMs) of 4F2hc-LAT2 are clearly visible as white intense lines from 2D-class average side-view projections (Figure 2B).

**Figure 3:**
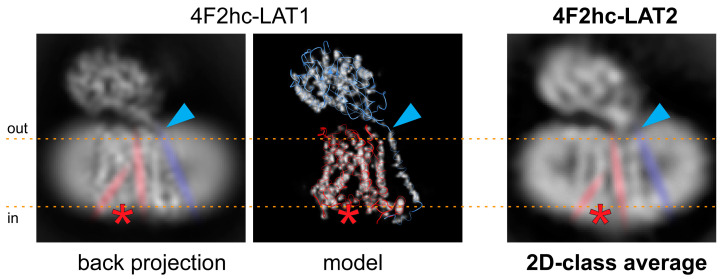
Comparison of the 4F2hc-LAT2 architecture with the back projected cryo-EM density map and the structural model of 4F2hc-LAT1. Individual representations of 4F2hc-LAT1/2 are oriented as viewed from the membrane plane and the membrane boundaries facing the extracellular (out) and cytoplasmic (in) environments are indicated with orange dotted lines. The red asterisks refer to the location of a gap toward the cytoplasm in the structures, which is the result of the inward-open architecture of 4F2hc-LAT1/2. The blue arrowheads highlight the location of the disulfide bridge between 4F2hc and LAT1/2. In addition, the 30° tilted straight density originating from TMs 1a, 6b, and 10, the vertical intense density originating from TMs 3 and 8, and the density from TM connecting the intracellular with the extracellular domain of 4F2hc (TM1–4F2hc) are indicated in red and blue, respectively. Note that both structural features discussed in 4F2hc-LAT1 are also present in side-view 2D-class average of 4F2hc-LAT2 (right).

Based on the 4F2hc-LAT1 cryo-EM volume (EMD-9721 [23]), which was lowpass filtered to 5 Å and back projections calculated using RELION [25], a similar conformation of our 4F2hc-LAT2 particles is suggested (Figure 3). The back projected cryo-EM density map of 4F2hc-LAT1 reveals a strong, vertical, and central density in the membrane-spanning portion. This density is accompanied on the left by a ≈30° tilted straight density, leaving a gap toward the cytoplasm between those two densities (discussed densities are colored in red, and the location of the gap is indicated with red asterisks in Figure 3). According to the structural model (PDB code 6IRS, Figure 3 image in de middle), the main contributions of these densities are identified to originate from amino acid residues located in TMs 3 and 8, and TMs 1a, 6b, and 10 of LAT1. TMs 1 and 6 of human LAT1, as well as of the bacterial homologue L-arginine/agmatine exchanger AdiC, and supposedly also of the human LAT2 are split into discontinuous, α-helical elements, i.e., TM1a, TM1b, TM6a, and TM6b [23,24,26,27]. Based on the rocking-bundle alternating-access mechanism of LeuT fold proteins, these discontinuous α-helical elements (i) interact extensively with the substrate and (ii) are supposed to move against each other during substrate transport [28,29]. In some of the 2D-class averages of 4F2hc-LAT2, the strong and kinked density resulting in a gap toward the cytoplasm is also present (Figure 2B and 3, rightmost image). Therefore, we propose a similar inward-open conformation for GDN-solubilized 4F2hc-LAT2 as previously reported for 4F2hc-LAT1 [23,30]. The TM connecting the intracellular with the extracellular domain of 4F2hc (TM1–4F2hc) is visible in the 2D-class average. The C-terminal end of TM1–4F2hc is the approximate location of the disulfide bridge between LAT1/2 and 4F2hc as indicated (Figure 3, blue arrowheads).

**Figure 4:**
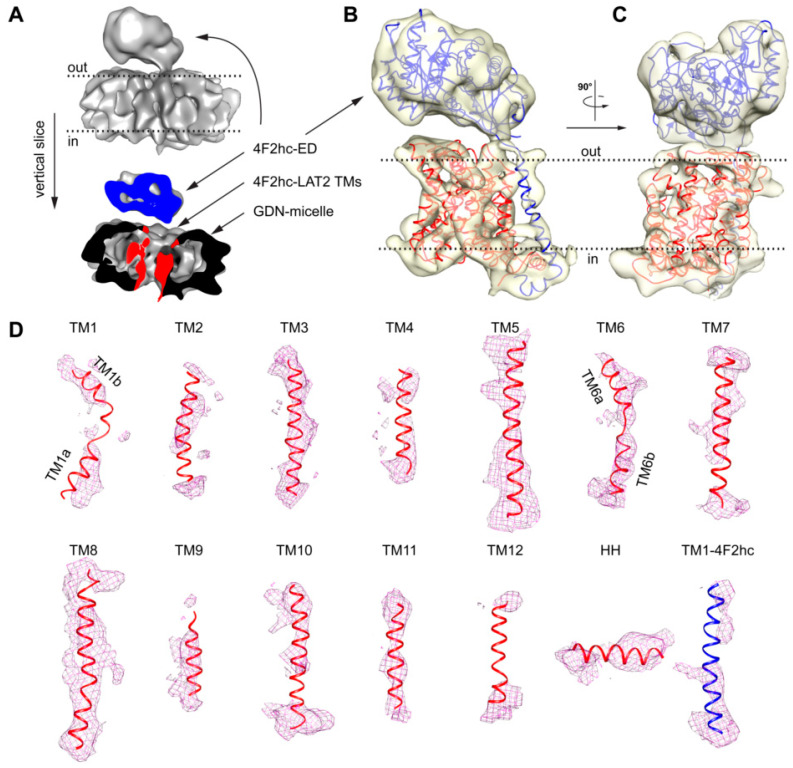
Cryo-EM 3D density map of 4F2hc-LAT2 and analysis of 4F2hc-LAT2^hom^ homology model fit. (**A**) The upper part represents the overall 4F2hc-LAT2 cryo-EM volume at a resolution of ≈7.5 Å. The lower part depicts a vertical slice through the 4F2hc-LAT2 volume displaying protein (blue, 4F2hc-ED; red, TMs of 4F2hc-LAT2) and micellar (black) density contributions. (**B**,**C**) Side views of the protein cryo-EM density displayed as a transparent yellowish surface and viewed from the membrane plane (**B**) and in-plane rotated by 90° (**C**). The 4F2hc-LAT2^hom^ model is fitted into the density map. (**D**) Shown are individual protein cryo-EM densities as magenta-colored meshes of TMs located in the GDN-detergent micelle as the TMs 1–12 and horizontal α-helix (HH) of LAT2, and TM1–4F2hc. The 4F2hc-LAT2^hom^ model fitted into the protein cryo-EM density is shown as a ribbon and colored in blue (4F2hc) and red (LAT2). Note that in panels (**A**–**C**), membrane boundaries facing the extracellular (out) and cytoplasmic (in) environments are indicated with black dotted lines, and 4F2hc-LAT2 volumes shown are oriented as viewed from the membrane plane. For panel (**D**), individual TMs are rotated such that the upper and lower parts are facing the extracellular and cytoplasmic environments, respectively.

Several rounds of 2D- and 3D-classification in RELION [25] and further ab initio 3D-model reconstruction applying cryoSPARC [31] were used to weed out “bad” particles (a data processing flow chart is given in Figure S1A). Finally, a set of 104,971 particles were used to calculate a cryo-EM volume at a resolution of approximately 7.5 Å (for data processing statistics and cryo-EM density analysis, see Table S1 and Figure S1B–D). The density showed a bilobed structure consisting of a smaller and a larger volume similar to the previously published 4F2hc-LAT2 volumes [5,21,22]. Whereas the smaller volume was identified as the 4F2hc-ectodomain (4F2hc-ED), the larger volume was attributed to the TMs of 4F2hc-LAT2 surrounded by a GDN micelle (Figure 4A). The larger volume was composed of mainly two connected domains, i.e., (i) a peripheral intense density originating from the GDN-detergent micellar headgroups and (ii) an inner, structured protein density (Figure 4A). This is common for detergent solubilized membrane protein cryo-EM volumes, since the hollow appearance between the micellar headgroup and the proteinaceous density is attributed to the hydrophobic portion of detergents [32]. Unlike in the structure of 4F2hc-LAT1 reported by Yan and colleagues [23], who also used GDN as a detergent for cryo-EM specimen preparation, the final volume of 4F2hc-LAT2 displayed a very strong micellar headgroup density (Figure 4A). This strong density may explain the resolution restriction, since its density contribution is overestimated and thus the alignment of the protein density in individual particles is suboptimal. Although LAT1 and LAT2 are based on the primary amino acid sequence and are ≈48% identical and ≈65% similar to each other, the resolution of GDN purified 4F2hc-LAT2 was restricted to 7.5 Å, which is in stark contrast to published structures of GDN purified 4F2hc-LAT1 resolved at ≈3.3 Å [23,24]. It is known that in micelles, the scattering of electrons is particularly strong for the large hydrophilic headgroup [32], such as in GDN. Consequently, the usage of GDN is considered not to be an optimal detergent choice for the high-resolution structure solution of 4F2hc-LAT2.

Based on the aforementioned proposed inward-open conformation of 4F2hc-LAT2 and the high amino acid sequence identity and similarity between LAT2 and LAT1, a homology model of 4F2hc-LAT2 (4F2hc-LAT2^hom^) was generated using the LAT1 coordinates as input (PDB code 6IRS chain B). The model was calculated by Swiss-MODEL [33] and verified by the structural comparison of a second homology model calculated by a different server (see Material and Methods, Section 4.4.). The main differences between these homology models are found in loop regions, whereas the register and architecture in the TMs are equal. Apart from the 12 predicted transmembrane domains of LATs, the cryo-EM structure of 4F2hc-LAT1 also revealed the presence of a horizontal α-helix (HH) in the C-terminal end of LAT1. A structural feature of LAT1 that was discussed to interact with 4F2hc and if deleted abolished the substrate transport function of the heterodimeric complex 4F2hc-LAT1 [23]. The homology model 4F2hc-LAT2^hom^ was fitted into the cryo-EM volume (Figure 4B,C). The 4F2hc-ED was best resolved (Figure 4B,C). In the membrane-spanning portion of 4F2hc-LAT2, almost all 13 TMs, i.e., the 12 LAT2 TMs and TM1–4F2hc, were also resolved (Figure 4B–D). In addition, the density for the C-terminal HH in LAT2 is also present (Figure 4D). As mentioned above, TMs 1a, 1b, 6a, and 6b are expected to move to a large extent upon transition from the inward-open to the outward-open state [23,24,26,27]. These domains are relatively well resolved in our cryo-EM volume of 4F2hc-LAT2, displaying the kinks of individual TMs (Figure 4D). In summary, the overall nice fit of 4F2hc-LAT2^hom^ to the cryo-EM density map of 4F2hc-LAT2 (Figure 4) strengthens the hypothesis that GDN purified 4F2hc-LAT2 adopts an inward-open conformational state.

**Figure 5:**
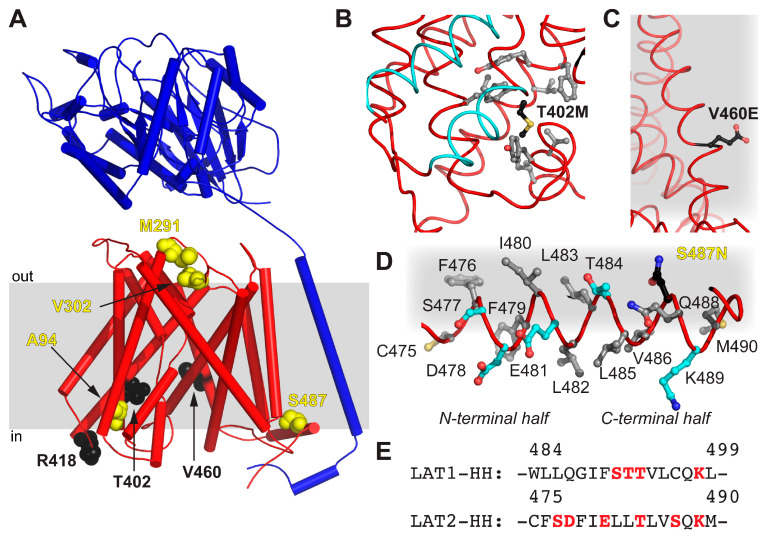
Mapping of disease-causing point mutations of human LAT2 on 4F2hc-LAT2^hom^. (**A**) Overall structure of 4F2hc-LAT2^hom^. The location of residues related with cataract formation (yellow spheres) and age-related hearing loss (black spheres) are shown. The heterodimeric complex is oriented inside the plasma membrane (gray bar) according to the PPM server [36] and shown as a ribbon representation (4F2hc, blue; LAT2, red). (**B**) Displayed are side chains of hydrophobic residues (gray sticks) within a distance of 4 Å to T402M (black sticks) and the substrate transport moving elements, i.e., TMs 1a and 6b (cyan). (**C**) Shown is V460E (black sticks) protruding into the hydrophobic portion of the plasma membrane (gray). (**D**) All side chains of residues including hydrophobic (gray), hydrophilic (cyan), and S487N (black sticks) of the horizontal α-helix HH of LAT2 are depicted. Note that the N-terminal half of the amphiphilic α-helix HH is correctly positioned at the cytosolic lipid interface (gray), whereas the C-terminal half is not. (**E**) Displayed is the primary amino acid sequence alignment between LAT1 and LAT2 in the HH region. Hydrophilic residues are highlighted in red. In panels (**B**–**D**), the LAT2 structure is displayed as ribbon and important residues are displayed as sticks.

The obtained homology model 4F2hc-LAT2^hom^ displays the currently most accurate model representing the structure of 4F2hc-LAT2 in the inward-open conformation. Therefore, 4F2hc-LAT2^hom^ was further used to map disease-causing point mutations of human LAT2, naturally occurring in human patients suffering from age-related hearing loss or cataract formation [14,15] (Figure 5A and Table 1). The point mutant 4F2hc-LAT2^M291I^ is involved in cataract formation, whereas 4F2hc-LAT2^V302I^ plays a role in both degenerative diseases. M291 and V302 of LAT2 are both located in an extracellular loop (EL) connecting TMs 7 and 8. In LAT1, this region is discussed to be rearranged upon substrate transport [24], and certain EL residues are involved in ionic interaction between 4F2hc and LAT1 [23]. The individual mutation of both residues, M291 and V302, to Ile had a negative effect on the transport of large amino acids (Trp and Tyr), but none on small ones (Ala and Ile) [14,15]. Our 4F2hc-LAT2^hom^ model supports the previous hypothesis that the point mutations 4F2hc-LAT2^M291I^ and 4F2hc-LAT2^V302I^ may alter EL flexibility due to steric reasons [14]. T402 is located in TM10, and its side chain is in close proximity to the substrate translocation path on the cytoplasmic side. The mutation T402M is hypothesized to alter the local hydrophobicity and may thus influence the structural flexibility upon substrate binding or transport (Figure 5B). If compared to the wild-type situation, the impaired structural flexibility is reflected by an almost 10-fold decrease in transport efficiency of 4F2hc-LAT2^T402M^ toward substrates Ala and Tyr [14]. On the other hand, V460 is located on TM12 of LAT2 and faces the lipidic environment (Figure 5C). The non-conservative mutation V460E alters the charge distribution of 4F2hc-LAT2^V460E^ on the lipid-facing surface of LAT2. Thus, hydrophobic interactions between LAT2 and the lipidic environment are disturbed. This may explain the overall 5-fold decrease in 4F2hc-LAT2^V460E^ expression and the fact that the protein complex did not reach the plasma membrane at all [14]. Furthermore, the presence of an HH in LAT2 is a novel finding (Figure 4D and Figure 5D). The analysis of LAT1-HH reveals a small hydrophilic part sandwiched by mostly hydrophobic stretches (Figure 5E). In contrast to LAT1-HH, the primary amino acid sequence of LAT2-HH shows a continuous distribution of hydrophilic and hydrophobic stretches throughout HH (Figure 5E), suggesting amphipathicity. Residues of the N-terminal region of LAT2-HH are positioned such that the amphipathic nature of LAT2-HH is discerned but residues in the C-terminal half are not (Figure 5D). Thus, we hypothesize that the α-helical register of HH in 4F2hc-LAT2^hom^ is not correct but rather shifted by one amino acid in C-terminal direction. Similar to this, the whole LAT2-HH would be correctly positioned to act as an amphipathic α-helix. Disruption of an amphipathic region may have an impact on protein/lipid interaction [34,35]. According to the 4F2hc-LAT2^hom^ model, S487 points toward the hydrophobic portion of the membrane as well as toward the protein moiety (Figure 5D) with no obvious hydrogen bonding partners nearby. Even if HH would adopt an alternate conformation as suggested above, a single point mutation in the C-terminal half of HH, e.g., 4F2hc-LAT2^S487N^, might not be enough to destabilize HH significantly. This is reflected by the unaltered Ala and Trp transport catalyzed by point mutant 4F2hc-LAT2^S487N^ into HeLa cells [15].

## 3. Conclusions

In this study, we have reported on the cryo-EM density map of the human 4F2hc-LAT2 amino acid exchanger resolved at sub-nanometer resolution. Although the protein sample was highly pure and monodisperse, the resolution was limited to 7.5 Å. Analysis of electron micrographs and final cryo-EM map revealed strong micellar headgroup density. The latter most probably leads to an improper alignment of individual particles and thus of the protein density limiting resolution. Moreover, we generated a homology model (4F2hc-LAT2^hom^) based on the 4F2hc-LAT1 structure [23]. The SEC purified human heterodimeric complex 4F2hc-LAT2 embedded in a GDN micelle revealed an inward-open conformation as analyzed by (i) a comparison of 2D-class averages of side views and back projection of the previously obtained cryo-EM volume of 4F2hc-LAT1 [23] and (ii) by fitting the inward-open 4F2hc-LAT2^hom^ model into the cryo-EM density of 4F2hc-LAT2. Furthermore, disease-causing point mutations of human LAT2 were mapped on the currently most accurate homology model of human 4F2hc-LAT2. In order to fully understand the architecture of 4F2hc-LAT2 and the molecular interaction network of disease-related mutations at a molecular level, a 4F2hc-LAT2 structure resolved at atomic resolution is indispensable. Thus, this work presents a further milestone toward this goal and paves the way for high-resolution structure solution by cryo-EM of the human 4F2hc-LAT2 complex.

## 4. Materials and Methods

### 4.1. Cloning, Overexpression, and Purification of Human 4F2hc-LAT2

The generation of recombinantly overexpressing human 4F2hc-LAT2 *Pichia pastoris* strains was reported previously [20]. The protein production procedure given in [22] was applied except that after induction, the incubation time was 48 h, instead of 28 h as previously reported. The cells were harvested by centrifugation at 10,000× *g*, 4 °C for 10 min, resuspended in 50 mM Na-phosphate pH 7.4, 10% (*v*/*v*) glycerol, 1 mM EDTA, and lysed by sonification (Brandson, Danbury, CT, USA) followed by 5 consecutive microfluidizer (Microfluidics, Westwood, MA, USA) cycles at 1500 bar. The cell debris were removed by a low-speed centrifugation run at 10,000× *g*, 4 °C for 10 min, and membranes were collected by ultracentrifugation at 150,000× *g*, 4 °C for 1 h. Crude membranes were washed by homogenization in 80 mM Bis-Tris propane pH 6.8, 300 mM NaCl, 10% (*v*/*v*) glycerol, and the washed membranes were collected by a second ultracentrifugation run at 150,000× *g*, 4 °C for 1 h. These membranes were homogenized, diluted with 80 mM Bis-Tris propane pH 6.8, 300 mM NaCl, 10% (*v*/*v*) glycerol to a concentration of approximately 300 mg membranes per mL, and finally flash frozen and stored at −75 °C until further use.

The human 4F2hc-LAT2 heteromeric complex was purified as reported previously by Ni-NTA affinity chromatography [5,20,21,22]. The heteromeric complex was eluted from the Ni-NTA resin with 20 mM Bis-Tris propane pH 6.8, 150 mM NaCl, 2% (*v*/*v*) glycerol, 0.02% (*w*/*v*) GDN, 10 mM oxidized gluthathione, and 200 mM L-histidine. The eluate was concentrated to ≈10 mg/mL with a Amicon 100-kDa molecular weight cut-off device (Merck, Switzerland). The concentrated sample was further purified by size exclusion chromatography (SEC) using a Superose 6 Increase 3.2/300 column and eluted with SEC-buffer (20 mM Bis-Tris propane pH 6.8, 150 mM NaCl, 0.02% (*w*/*v*) GDN, 5 mM L-histidine). Peak fractions at ≈3 mg/mL were collected and either directly used for cryo-EM grid preparation or further protein analysis by SEC and SDS-PAGE.

### 4.2. Grid Preparation and Cryo-EM Data Collection

After the final protein purification step by SEC, 3 µL of the 4F2hc-LAT2 sample at ≈3 mg/mL in SEC-buffer were adsorbed on glow discharged (20 s, 10 mA, 0.25 mbar) cryo-EM grids (Cu-R1.2/1.3 grids 300 Mesh; Quantifoil, Germany) for 3 s. The grids were plunge frozen in liquid ethane after blotting off excess buffer for 3 s using a Vitrobot Mark IV apparatus operated at approximately 100% humidity and cooled to 4–5 °C. The grids were stored in liquid nitrogen until further use. Cryo-EM data were collected using a 300 kV Thermo Fisher Scientific Titan Krios G4 electron microscope equipped with a Falcon 4 direct electron detector in an aberration-free image shifting and fringe-free illumination mode. The data were collected at a magnification of 96,000× corresponding to 0.83 Å/pix on the camera and at defocus ranges of −0.9 to −2.2 µm. Images were recorded in an automated fashion using the EPU 2 software (Thermo Fisher Scientific, Hillsboro, OR, USA) in counting mode for 4.6 s with a dose rate of 8.7 e^−^/Å^2^/s, resulting in a total accumulated dose on the specimen level of approximately 40 e^−^/Å^2^ per exposure.

### 4.3. Calculation of 2D-Class Averages and Cryo-EM Density Map

Dose-fractioned images were motion-corrected, and frames were dose-weighted using the software implemented in RELION 3.1 [25]. The contrast transfer function (CTF) parameters were estimated by ctffind 4.1.13 [37]. Images of insufficient quality, e.g., worse CTF-resolution than 5 Å, strong drift, and ice contamination were deselected manually using cryoSPARC [31]. From the remaining 3120 images, an initial set of 957,188 particles were picked using WARP [38] and used for image processing with RELION 3.1 and cryoSPARC 2.15. After several rounds of 2D and 3D particle classification using RELION 3.1, a remaining particle set of 151,306 particles were exported to cryoSPARC 2.15 and subjected to further classification rounds using the ab initio 3D-reconstruction algorithm by cryoSPARC. The remaining 104,971 particles served as input to calculate the final volume using cryoSPARC. Based on the Fourier shell correlation (FSC) cut-off criteria of 0.143 (gold standard) [39,40], the resolution was determined to 7.5 Å (Figure S1B). Data acquisition parameters and data processing information is given in Table S1. A data processing flow chart, FSC curve, local resolution estimation, and an angular distribution for particle projections are displayed in Figure S1. The cryo-EM map of 4F2hc-LAT2 in detergent has been deposited in the Electron Microscopy Data Bank under accession numbers EMD-11726.

### 4.4. Sequence Alignment Calculation of LATs and Homology Model Generation of 4F2hc-LAT2^hom^

The pairwise primary amino acid sequence alignment between human LAT1 (UniProt ID: Q01650) and human LAT2 (UniProt ID: Q9UHI5) was calculated by employing the web application EMBOSS needle (https://www.ebi.ac.uk/Tools/psa/emboss_needle/ [41]), and the sequence identity and similarity were determined to be 48% and 65%, as indicated above.

The homology model of 4F2hc-LAT2^hom^ was generated by applying the web application Swiss-MODEL (https://swissmodel.expasy.org/interactive; [33]) in an automated fashion using the amino acid sequence of human LAT2 (UniProt ID: Q9UHI5) as input. The best model was built based on the coordinates of the cryo-EM structure of 4F2hc-LAT1 (PDB code 6IRS chain B). The resulting LAT2 homology model (LAT2^hom^) was structurally aligned to LAT1 (PDB code 6IRS chain B) using the alignment tool of PyMOL (Molecular Graphics System Version 2.3.0, Schrodinger, LLC) resulting in an RMSD of 0.110 Å for 434 Cα-atoms. In order to increase the confidence in the register and the TM architecture of this Swiss-MODEL based LAT2 homology model, homology model calculation employing Phyre2 [42] was performed, which resulted in an RMSD of 0.541 Å for 412 Cα-atoms. Finally, the coordinates of 4F2hc (PDB code 6IRS chain A) and LAT2^hom^ (Swiss-MODEL) were combined, and the homology model of 4F2hc-LAT2 (4F2hc-LAT2^hom^) was saved as a PDB file.

### 4.5. Figure Preparation

Molecular graphics were performed with UCSF Chimera [43] and PyMOL (Molecular Graphics System Version 2.3.0, Schrodinger, LLC).

## Figures and Tables

**Table 1:** Structure-based potential implications of disease-causing point mutations of the human 4F2hc-LAT2.

Mutation	Location	Possible Implication
A94T	TM2	ne ^1^
M291I	EL ^2^ TM7–8	Alteration of EL flexibility due to steric reasons
V302I	EL ^2^ TM7–8	Alteration of EL flexibility due to steric reasons
T402M	TM10	Impaired structural flexibility due to local hydrophobicity alteration
R418C	CL ^3^-TM10–11	ne ^1^
V460E	TM12	Disturbance of LAT2 hydrophobic interactions with lipidic environment
S487N	HH	Contribution to the disruption of the amphipathic nature of HH

^1^ ne: No explanation.; ^2^ EL: Extracellular loop; ^3^ CL: Cytoplasmic loop.

## Supplementary Materials

Supplementary Materials can be found at https://www.mdpi.com/1422-0067/21/19/7094/s1.

## Abbreviations

3D	three-dimensional
4F2hc-LAT2T^hom^	homology model of 4F2hc-LAT2
CHS	cholesteryl hemisuccinate
CL	cytoplasmic loop
CTF	contrast transfer function
ED	ectodomain
EL	extracellular loop
EM	electron microscopy
EMD	electron microscopy databank
GDN	glyco-diosgenin
HH	horizontal α-helix
HAT	heterodimeric amino acid transporter
LAT	L-type amino acid transporter
LMNG	lauryl maltose neopentyl glycol
MW	molecular weight
PDB	protein databank
PPM	positioning of proteins in membrane
RMSD	root-mean-square deviation
SDS-PAGE	SDS-polyacrylamid gel electrophoresis
SEC	size exclusion chromatography
TM	transmembrane helix
UniProt-ID	protein knowledgebase-identifier
