# Supplementary material for: Sub-Nanometer Cryo-EM Density Map of the Human Heterodimeric Amino Acid Transporter 4F2hc-LAT2
Source: Int J Mol Sci. 2020 Sep 25;21(19):7094. doi: 10.3390/ijms21197094 (PMC7584034; doi:10.3390/ijms21197094)
Supplement: Supplementary file 1 [file ijms-21-07094-s001.pdf]

Supplementary Material

to

Sub-nanometer cryo-EM density map
of the human heterodimeric amino acid transporter 4F2hc-LAT2

by

J.-M. Jeckelmann and D. Fotiadis

Table 1. Data acquisition and processing information.

**Electron Microscope.**	**Thermo Scientific Titan Krios G4**
Direct electron detector camera	Falcon 4
Data acquisition software	EPU 2
Magnification	96,000 x
Magnification voltage (kV)	300
Electron exposure ($e^-/Å^2$)	40
Dose rate ($e^-/Å^2/s$)	8.7
Acquisition time per image (s)	4.6
Defocus range (μm)	-0.9 to -2.2
Pixel size (Å/pix)	0.83
Symmetry imposed	C1
Initial electron micrographs (no.)	5,967
Final electron micrographs (no.)	3,120
Initial particle images (no.)	957,188
Final particle images (no.)	104,971
Map resolution (Å)	7.5
FSC threshold	0.143

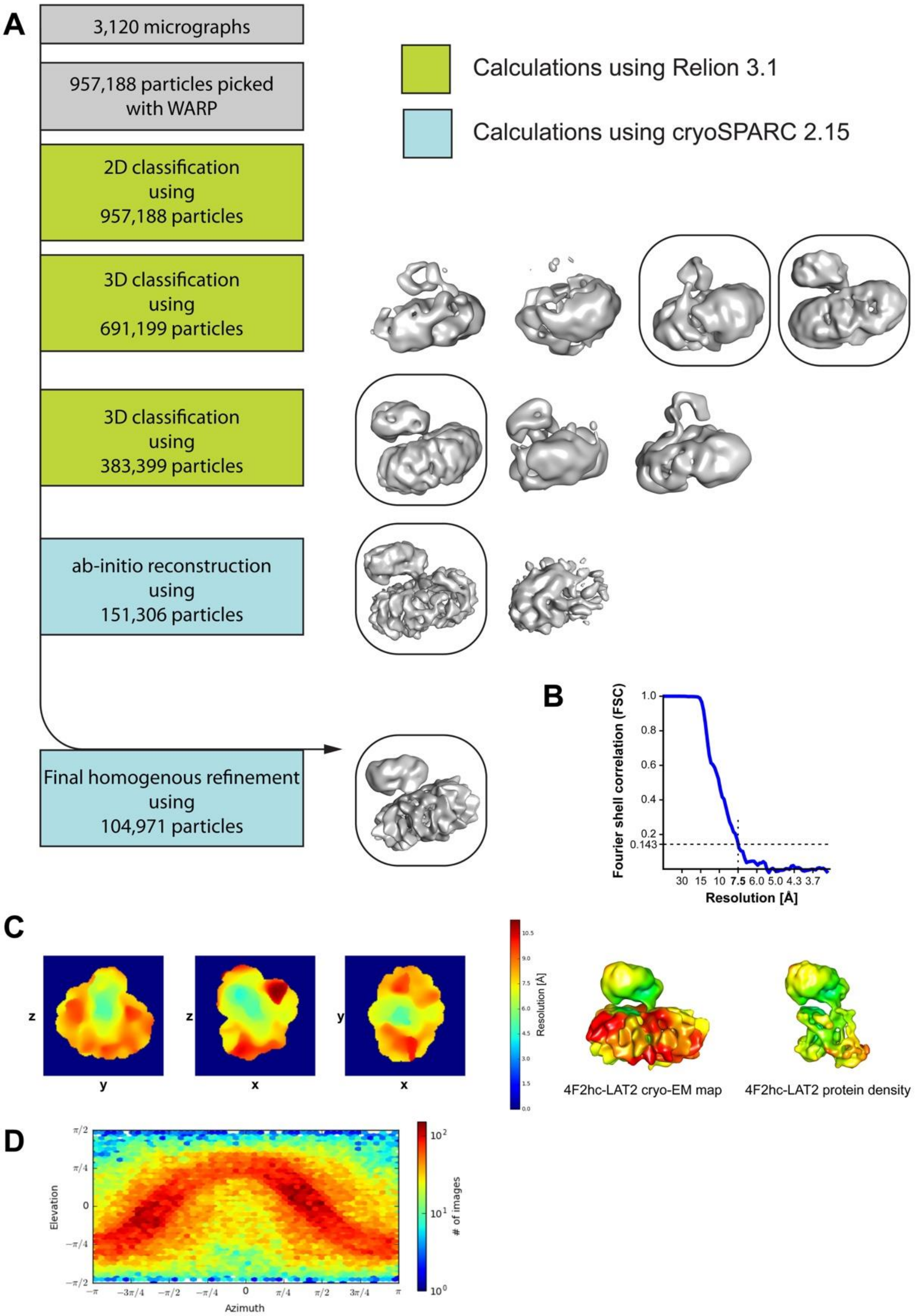

Figure S1. Flow chart for cryo-EM data processing and cryo-EM map analysis. (**A**) Displayed are details of the individual cryo-EM data processing steps as given in the Materials and Methods section. Calculations performed using Relion and cryoSPARC are highlighted in green and light blue, respectively. The cryo-EM maps obtained are shown and those selected and used for a next calculation step are boxed. (**B**) The Fourier shell correlation (FSC) plot for the calculated 4F2hc-LAT2 density map indicated a resolution of 7.5 Å at a cut-off 0.143 (gold standard) criterion. (**C**) Local resolution map calculated using the final cryo-EM map of 4F2hc-LAT2 by cryoSPARC. Whereas on the left-hand side central slices through the map are displayed, on the right side 3D representations of the cryo-EM of map plus the protein density are shown. (**D**) Angular distribution for particle projections calculated using cryoSPARC. The heat map displays the number of particles for each viewing angle.
